# Blockage of the Ryanodine Receptor via Azumolene Does Not Prevent Mechanical Ventilation-Induced Diaphragm Atrophy

**DOI:** 10.1371/journal.pone.0148161

**Published:** 2016-02-05

**Authors:** Erin E. Talbert, Ashley J. Smuder, Oh Sung Kwon, Kurt J. Sollanek, Michael P. Wiggs, Scott K. Powers

**Affiliations:** Department of Applied Physiology and Kinesiology, University of Florida, Gainesville, Florida, United States of America; Faculty of Animal Sciences and Food Engineering, University of São Paulo, BRAZIL

## Abstract

Mechanical ventilation (MV) is a life-saving intervention for patients in respiratory failure. However, prolonged MV causes the rapid development of diaphragm muscle atrophy, and diaphragmatic weakness may contribute to difficult weaning from MV. Therefore, developing a therapeutic countermeasure to protect against MV-induced diaphragmatic atrophy is important. MV-induced diaphragm atrophy is due, at least in part, to increased production of reactive oxygen species (ROS) from diaphragm mitochondria and the activation of key muscle proteases (i.e., calpain and caspase-3). In this regard, leakage of calcium through the ryanodine receptor (RyR1) in diaphragm muscle fibers during MV could result in increased mitochondrial ROS emission, protease activation, and diaphragm atrophy. Therefore, these experiments tested the hypothesis that a pharmacological blockade of the RyR1 in diaphragm fibers with azumolene (AZ) would prevent MV-induced increases in mitochondrial ROS production, protease activation, and diaphragmatic atrophy. Adult female Sprague-Dawley rats underwent 12 hours of full-support MV while receiving either AZ or vehicle. At the end of the experiment, mitochondrial ROS emission, protease activation, and fiber cross-sectional area were determined in diaphragm muscle fibers. Decreases in muscle force production following MV indicate that the diaphragm took up a sufficient quantity of AZ to block calcium release through the RyR1. However, our findings reveal that AZ treatment did not prevent the MV-induced increase in mitochondrial ROS emission or protease activation in the diaphragm. Importantly, AZ treatment did not prevent MV-induced diaphragm fiber atrophy. Thus, pharmacological inhibition of the RyR1 in diaphragm muscle fibers is not sufficient to prevent MV-induced diaphragm atrophy.

## Introduction

Mechanical ventilation (MV) is a clinical intervention used to maintain adequate pulmonary gas exchange in patients who are unable to maintain adequate alveolar ventilation on their own. Common indications for MV include respiratory failure, heart failure, coma, and drug overdose. While MV can be a life-saving intervention, extended periods of MV lead to diaphragm atrophy and contractile dysfunction, collectively termed ventilator-induced diaphragm dysfunction (VIDD) [1–3]. MV-induced inspiratory muscle weakness is an important clinical problem, as VIDD is predicted to contribute to problems in weaning patients from MV. Indeed, approximately 25% of all intensive care patients experience difficult weaning, and difficult weaning leads to prolonged hospital stays along with increased morbidity and mortality in patients [4–6].

Although the mechanism(s) responsible for the development of VIDD remain an active area of research, recent studies reveal that an MV-induced increase in reactive oxygen species (ROS) production in the diaphragm is required for the development of VIDD, and the major source of ROS during prolonged MV appears to be of mitochondrial origin [7–11]. Increased mitochondrial ROS emission leads to activation of the key proteases calpain and caspase-3, which are also essential for the development of VIDD [10,12]. Due to the robust activation of the calcium-activated protease calpain, it is probable that prolonged MV results in increased levels of free calcium in the cytosol of diaphragm muscle fibers, which is required to activate calpain [13–15]. One possible source of increased cytosolic calcium in the diaphragm during prolonged MV is the leakage of calcium through the ryanodine receptor (RyR1). The RyR1 is the gated calcium channel located on the sarcoplasmic reticulum (SR) [16]; this channel generally remains closed unless stimulated to open by a nerve impulse, leading to calcium release from the SR and muscle contraction. However, oxidation of the RyR1 can result in calcium leak into the cytosol and recent evidence suggests that leaky RyR1s contribute to age-induced sarcopenia, muscular dystrophy, and skeletal muscle dysfunction due to heart failure [17–20]. In animal models of each of these pathologies, muscle function is improved by treatment with a compound that stabilizes the RyR1 and reduces calcium release [17]. Further, evidence indicates that treatment with dantrolene, a RyR1-blocking molecule, prevents sepsis-induced calpain expression and muscle breakdown [21].

It is also possible that RyR1 leak may contribute to the increased mitochondrial ROS production during prolonged MV. Indeed, increases in cytosolic calcium lead to increases in calcium content of the mitochondrial matrix because of the charge differential between the cytosol and mitochondrial matrix [22]. Increased mitochondrial calcium content stimulates several Krebs cycle enzymes and increases electron flow through the electron transport chain (ETC) [23,24]. During periods of muscle contractions, increased mitochondrial calcium increases mitochondrial production of ATP. However, during prolonged inactivity, limited ADP is available and the rate of oxidative phosphorylation declines. Because the electrical potential across the mitochondrial membrane is not being used to produce ATP, the energy potential continues to rise, and can lead to increased electron leak and elevated mitochondrial ROS production [23,25–27].

Therefore, these experiments tested the hypothesis that RyR1 leak in diaphragm muscle fibers is required for MV-induced increases in mitochondrial ROS emission, protease activation, and diaphragmatic atrophy. Cause and effect was determined by pharmacological blockade of the RyR1 in the diaphragm during 12 hours of MV. Our results reveal that blockade of the RyR1 is not sufficient to prevent MV-induced diaphragm mitochondrial ROS production, protease activation, or diaphragm atrophy.

## Materials and Methods

### Animals

Young adult female Sprague-Dawley rats (Harlan Laboratories, Indianapolis, IN) were housed by University of Florida Animal Care Services. Animals received standard rat chow and water ad libitum. All experiments were conducted in accordance with the Guide for the Care and Use of Laboratory Animals and approved by the University of Florida Institutional Animal Care and Use Committee (Protocol # 201105978).

### Experimental Protocol

Rats were randomly assigned to one of four experimental groups (n = 8-10/group): 1) Control-Vehicle (CON-VEH), 2) Control-Azumolene (CON-AZ), 3) MV-Vehicle (MV-VEH) and 4) MV-Azumolene (MV-AZ). We selected azumolene (AZ) to provide a pharmacological blockade of the RyR1 in the diaphragm because this compound is water-soluble and provides effective blockade of skeletal muscle RyR1 that successfully prevents local SR calcium release events (i.e., calcium sparks) [28,29].

Animals were anesthetized with an intraperitoneal injection of sodium pentobarbital (60 mg/kg). Once animals reached a surgical plane of anesthesia, an incision was made in the neck, allowing access to the trachea, a carotid artery, and a jugular vein. The jugular vein was cannulated and either azumolene sodium (10 mg/kg) or vehicle (90% saline, 10% dimethylsulfoxide) was infused. Azumolene sodium has previously been used *in vivo* in rats without apparent toxicity, with a dose of 10 mg/kg being sufficient to decrease muscle twitch force production by approximately 60% [30].

Following infusion of azumolene (AZ) or vehicle, animals were tracheostomized. Immediately following tracheostomy, animals in the control groups (CON-VEH and CON-AZ) had both solei removed and then were sacrificed by removal of the heart and diaphragm. Animals in the MV groups (MV-VEH and MV-AZ) underwent a tracheostomy and 12 hours of prolonged MV. MV animals received a constant infusion of vehicle (10% DMSO, 90% saline) over the 12 hours of MV, while MV-AZ animals received 0.83 mg/kg/hr sodium azumolene. The MV-VEH and MV-AZ groups were sacrificed in the same manner as control animals at the conclusion of 12 hours of MV.

### Details of MV

Ventilator settings (Servo Ventilator 300, Siemens) were as follows: upper airway pressure limit: 20 cm H_2_O, typical pressure generation above PEEP: 6–9 cm H_2_O, respiratory rate: 80 breaths/min; and PEEP: 1 cm H_2_O. Animals were constantly monitored during MV. Continuing care included expressing the animals’ bladders, rotating the animals to prevent blood pooling, and suctioning the airway to prevent mucus plugs. At the start of MV, animals received an intramuscular injection of glycopyrrolate (0.08 mg/kg) to decrease airway mucus secretions during MV. Animals also received a maintenance dose of glycopyrrolate (0.04 mg/kg) every two hours. Body temperature was read by a rectal thermometer and maintained at 37°C by resting animals on a recirculating water blanket. Heart rate was monitored by electrocardiograph. Following the initiation of MV, the carotid artery was cannulated to measure blood pressure. Blood samples (~100 μL) were taken from this catheter to ensure blood gas homeostasis was maintained during MV. If blood gas parameters changed, either tidal volume and/or oxygen concentration of the inspired air was increased to return to homeostasis. To maintain a surgical plane of anesthesia, sodium pentobarbital diluted in saline was constantly infused into a catheter in the jugular vein (~10 mg/kg/hr).

### Tissue Harvesting

The costal diaphragm was removed and divided into several sections, including one to measure contractile function, one to measure ROS emission, and one stored for histochemistry. The remaining diaphragm was quickly frozen in liquid nitrogen and stored at -80°C for analysis by western blotting. Additionally, one intact soleus muscle was used to measure contractile function.

### Functional Measurements

*In vitro* contractile function was measured in one intact soleus muscle and one ~25 mg strip of diaphragm muscle. Diaphragm function was measured as previously described, with the exception that the optimal length for muscle force production was not determined in the animals treated with AZ [31]. Specifically, because treatment of animals with AZ impairs calcium release from the RyR1 in response to electrical stimulation, it is not possible to determine the muscle’s optimal length for force production in animals treated with AZ. Therefore, after determining that the mean baseline diaphragm tension was 2 grams when the optimal diaphragm functional length was determined in our control animals (i.e., VEH group), we established a baseline tension of 2 grams for all diaphragm strips in this study. After establishing a baseline tension of 2 grams, 3 maximal tetanic contractions were performed followed by the measurement of an isometric force frequency curve as previously described [31]. Muscle force production was normalized to muscle cross sectional area using the method described by Segal [32].

Soleus muscle force production was measured in the same manner as diaphragm force production. Muscle optimal length was again set at the VEH average of 20 g of baseline force. Soleus force production was normalized to the muscle wet weight.

### Measurement of Mitochondrial ROS Production

#### Permeabilization of diaphragm fiber bundles

Small strips of diaphragm were permeabilized according to the method of Anderson [33]. Briefly, a ~10 mg piece of diaphragm was teased apart to near-single fibers on ice in buffer X (60 mM K-MES, 35 mM KCl, 7.23 mM K_2_EGTA, 2.77 mM CaK_2_EGTA, 20 mM imidazole, 0.5 mM DTT, 20 mM taurine, 5.7 mM ATP, 15 mM phosphocreatine, and 6.56 mM MgCl_2_, pH 7.1). Fiber bundles were permeabilized using Buffer X containing 100 μg/ml saponin for 30 minutes while rotating at 4°C. Fiber bundles were then washed three times for five minutes each in buffer Z (110 mM K-MES, 35 mM KCl, 1 mM EGTA, 5 mM K_2_HPO_4_, 3 mM MgCl_2_, 0.05 mM glutamate, 0.02 mM malate, and 0.5 mg/ml BSA, pH 7.1).

#### Mitochondrial ROS emission

Diaphragmatic mitochondrial ROS emission from a permeabilized fiber bundle was determined using the Amplex Red^TM^ reagent (Life Technologies). Details of this assay have been described previously [11]. During this assay, diaphragm fiber bundles are incubated with succinate, creatine kinase, creatine phosphate, and creatine to allow the mitochondria to respire; this approach produces a reliable physiological measurement of mitochondrial ROS production. Sample fluorescence was measured after 15 minutes of incubation at 37°C. The fluorescence was normalized to dry weight of the tissue to control for the size of the muscle fiber bundle.

### Histological Measurements

One piece of diaphragm was embedded in Optimal Cutting Temperature media (Sakura Fintek USA, Torrance, CA) and frozen in liquid nitrogen-cooled isopentane. Sections from these diaphragm samples (10 μm) were cut using a cryotome (ThermoScientific) and stained for dystrophin, myosin heavy chain (MHC) I and MHC type IIA proteins for fiber cross-sectional area analysis (CSA) as described previously [34]. CSA was determined using Scion Image software (Scion Corp., Frederick MD).

### Western Blot Analysis

Diaphragm tissue pieces were homogenized in 5 mM Tris, 5 mM EDTA buffer 10:1 (weight:volume) containing protease inhibitor cocktail (1:20 vol/vol, Sigma-Aldrich) using a motorized glass-on-glass system. Protein concentration was determined using the Bradford method [35] and muscle samples were normalized to a standard protein concentration. Following normalization, abundance of specific proteins was determined in diaphragm samples via western blotting using previously described methods [34]. The antibody against calpain (#2556, dilution: 1:500) was from Cell Signaling and the antibody against α-II-spectrin (sc-48382, 1:200) was from Santa Cruz Biotechnology. Protein abundances were normalized to α-tubulin (Santa Cruz Biotechnology), which served as a loading control.

### Measurement of Caspase-3 Activity

Caspase-3 activity was measured using the Caspase-Glo assay system (Promega), as previously described by our group [36]. Fifty μg of total protein was utilized to measure bioluminescent caspase-3 activity.

### Statistical Analysis

Comparisons between groups for each dependent variable were made by a two-way analysis of variance (ANOVA), using MV/CON and VEH/AZ as the factors. When appropriate, a Bonferroni test was performed post-hoc. Comparisons of blood gas parameters were made using at two-tailed Student’s t test. Significance was established *a priori* at α < 0.05.

## Results

### Biological Response to MV

No significant differences existed in body weight between the groups (Table 1). Treatment with AZ during prolonged MV did not cause any significant differences in mean arterial blood pressure, heart rate, PaCO_2_, PaO_2_, blood pH, or blood calcium concentrations compared to MV animals receiving VEH only during the course of 12 hours of MV (Table 2). No animal exhibited any indication of infection or significant lung injury following 12 hours of MV.

**Table 1:** Body Weights (g ± SEM) n = 8-10/group.

	CON	MV
VEH	269 ± 2	269 ± 3
AZ	270 ± 5	272 ± 4

**Table 2:** Arterial blood pressure and blood gas measurements (mean ± SEM) at the completion of the 12-hour experiment (n = 8-10/group).

	MV-VEH	MV-AZ
Arterial Blood Pressure (mm Hg)	101 ± 3	101 ± 4
Heart Rate (beats per minute)	343 ± 7	345 ± 6
Arterial pCO2 (torr)	36 ± 1	37 ± 1
Arterial pO2 (torr)	78 ± 3	70 ± 3
pH	7.43 ± 0.01	7.40 ± 0.01
Ca^2+^ (mmol/L)	0.98 ± 0.03	0.98 ± 0.05

### Blockade of RyR1 by AZ reduces force production in diaphragm muscle

**Fig 1:**
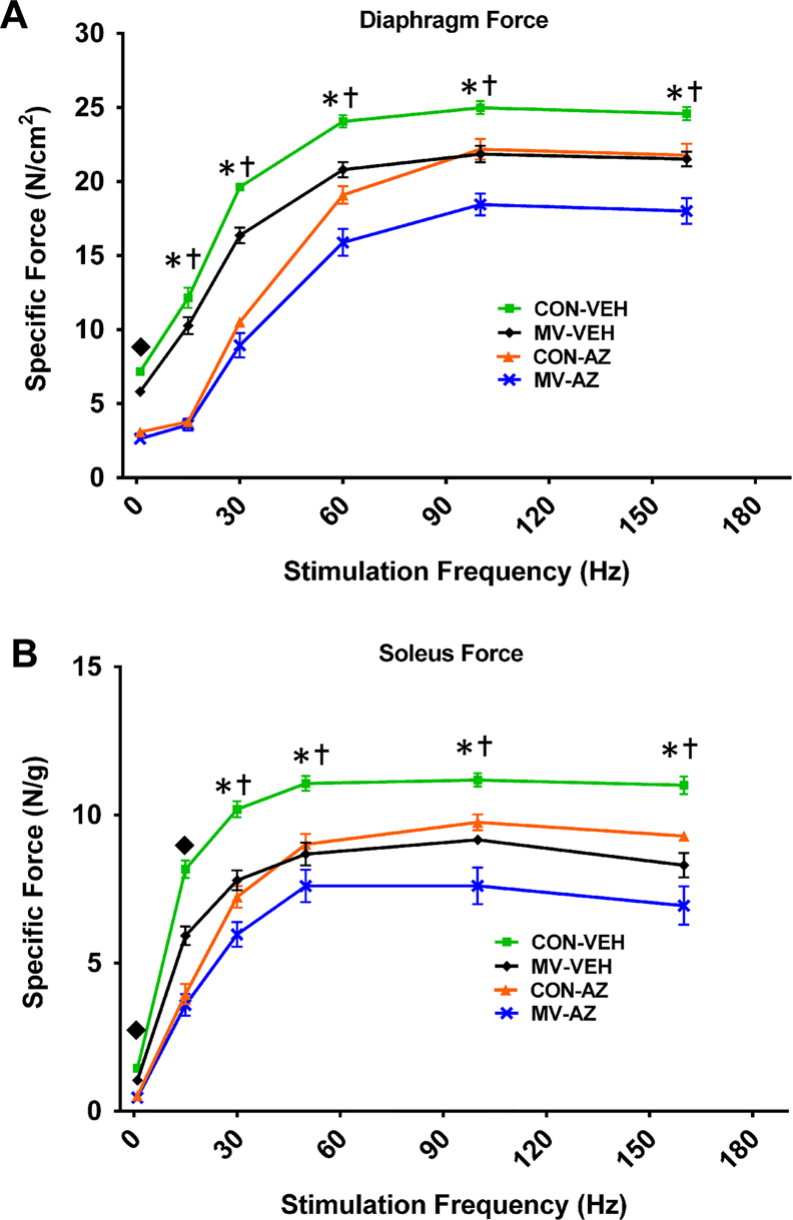
Muscle Contractile Function. Specific force was measured in (a) strips of diaphragm muscle and (b) the intact soleus muscle. Values are means ± SEM. * = main effect for MV (*p* < .05), = main effect for AZ (*p* < .05), ω = interaction effect between MV and AZ (*p* < .05), n = 8-10/group.

To confirm the presence of AZ within the diaphragm following 12 hours of MV, we measured the electrically stimulated *in vitro* specific force production in strips of diaphragm muscle (Fig 1A) and intact soleus muscles (Fig 1B) at stimulation frequencies ranging from 15Hz to 160Hz. AZ administration significantly decreased diaphragm and soleus muscle force production independent of MV. As previously described, 12 hours of MV significantly decreased the specific force produced by the diaphragm [9,12,37]. Additionally, 12 hours of MV significantly decreased soleus muscle specific force production.

### MV Promotes Mitochondrial ROS Production and Protease Activation in Diaphragm Muscle Fibers

**Fig 2:**
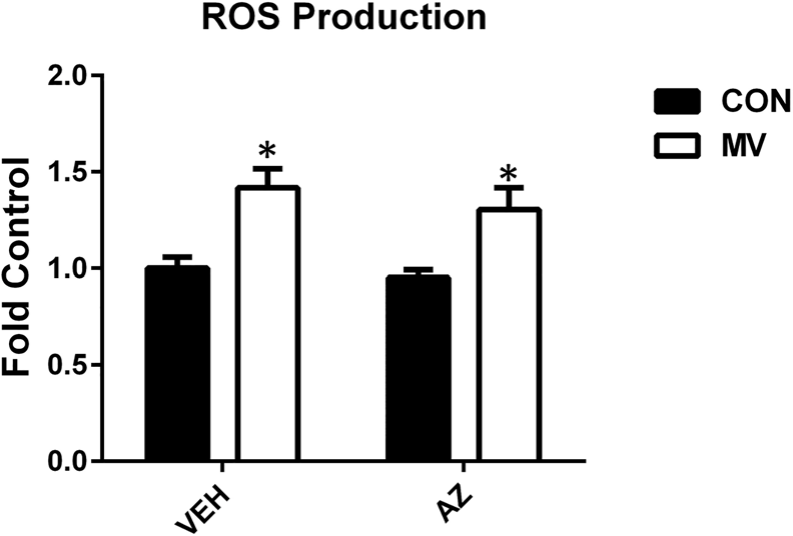
Reactive oxygen species emission. Hydrogen peroxide (H_2_O_2_) release from permeabilized diaphragm fibers. Values are presented as fold control of mean arbitrary units normalized to muscle dry weight ± SEM. * = main effect for MV (*p* < .05), n = 7-10/group.

It is established that MV significantly increases ROS emission from diaphragm mitochondria, but the mechanism for this increase remains unknown [10,11]. Blockade of the RyR1 with AZ was not sufficient to prevent the MV-induced increase in mitochondrial ROS emission (Fig 2). This finding suggests that the MV-induced increase in mitochondrial ROS production in diaphragm muscle fibers is not caused by leakage of calcium through the RyR1.

**Fig 3:**
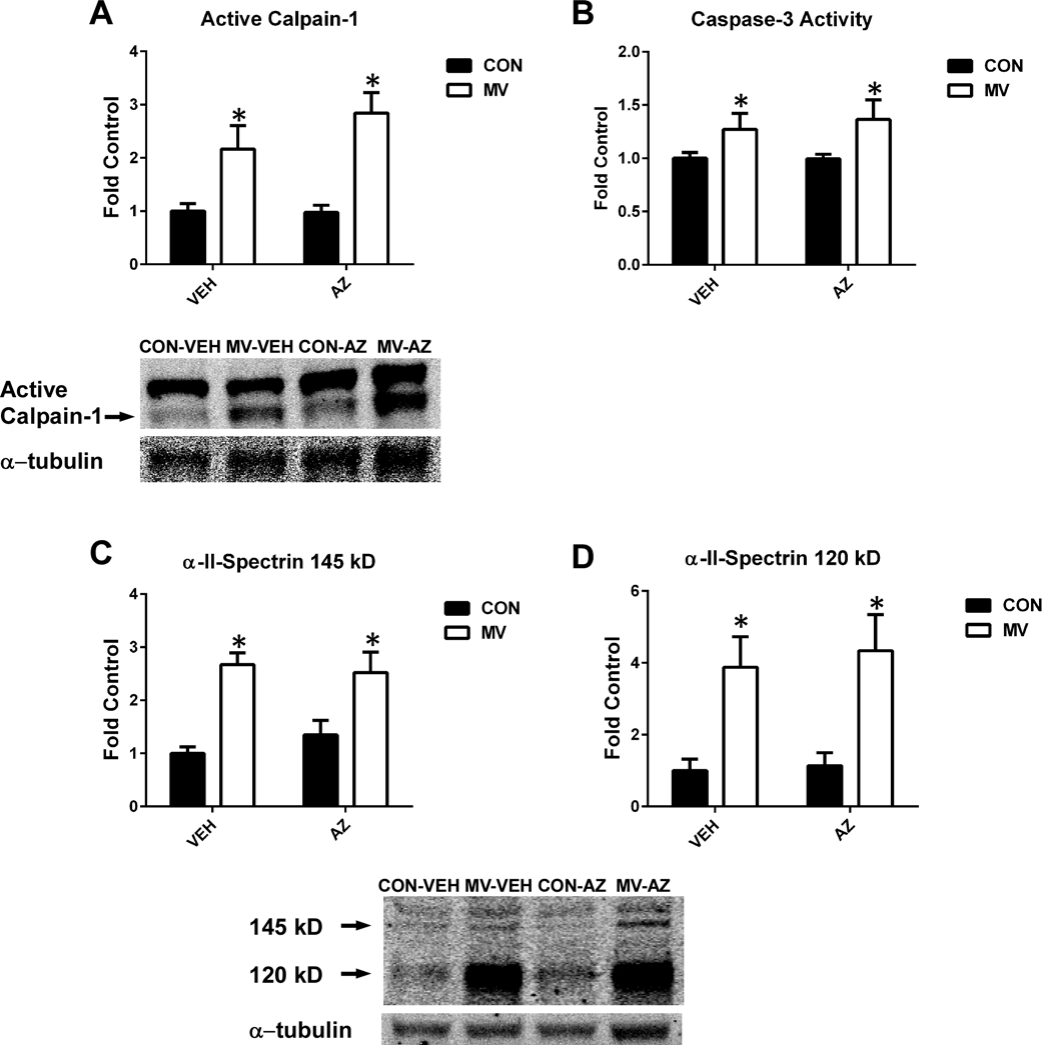
Activity of calpain and caspase-3. A) Levels of active calpain-1 were detected by Western blot. B) Caspase-3 activity. C) Specific α-II-spectrin cleavage product of calpain (145 kD) was detected via Western blot, D) Specific α-II-spectrin cleavage product of caspase-3 (120 kD) was detected by Western blot. Values are presented as fold control of mean arbitrary units ± SEM. Blots were normalized to α-tubulin. * = main effect for MV (*p* < .05), n = 6/group.

Activation of both calpain and caspase-3 in the diaphragm is required for the development of VIDD [12,34], and increases in the active form of both proteases in the diaphragm is well-documented following 12 hours of MV [9,10,12,38]. It is established that increased cytosolic levels of free calcium is required to activate calpain in cells [39]. If the MV-induced increase in cytosolic calcium levels in diaphragm fibers is due to calcium release from the RyR1, we predicted that blockade of the RyR1 would attenuate calpain activation in the diaphragm. However, our results do not support this hypothesis, as AZ treatment did not attenuate the MV-induced increases in calpain-1 activation (Fig 3A and 3C). This finding indicates that RyR1 blockade with AZ does not prevent the MV-induced increase in cytosolic calcium in the diaphragm.

In addition to the activation of calpains, calcium release via the RyR1 may also contribute to the activation of caspase-3 in the diaphragm during prolonged MV. Moreover, a regulatory cross-talk exists between calpain and caspase-3 in the diaphragm during prolonged MV, whereby the activation of calpain promotes caspase-3 activation and vice-versa [12]. Similar to calpain, we hypothesized calcium release from the RyR1 may contribute to caspase-3 activation in the diaphragm during prolonged MV. Identical to the findings with calpain, prolonged MV resulted in a significant increase in caspase-3 activity in the diaphragm, and pharmacological blockade of the RyR1 did not prevent MV-induced caspase-3 activation (Fig 3B and 3D). Representative images appear in Fig 3, with complete blots in S1 File.

### MV-induced Diaphragm Muscle Atrophy

**Fig 4:**
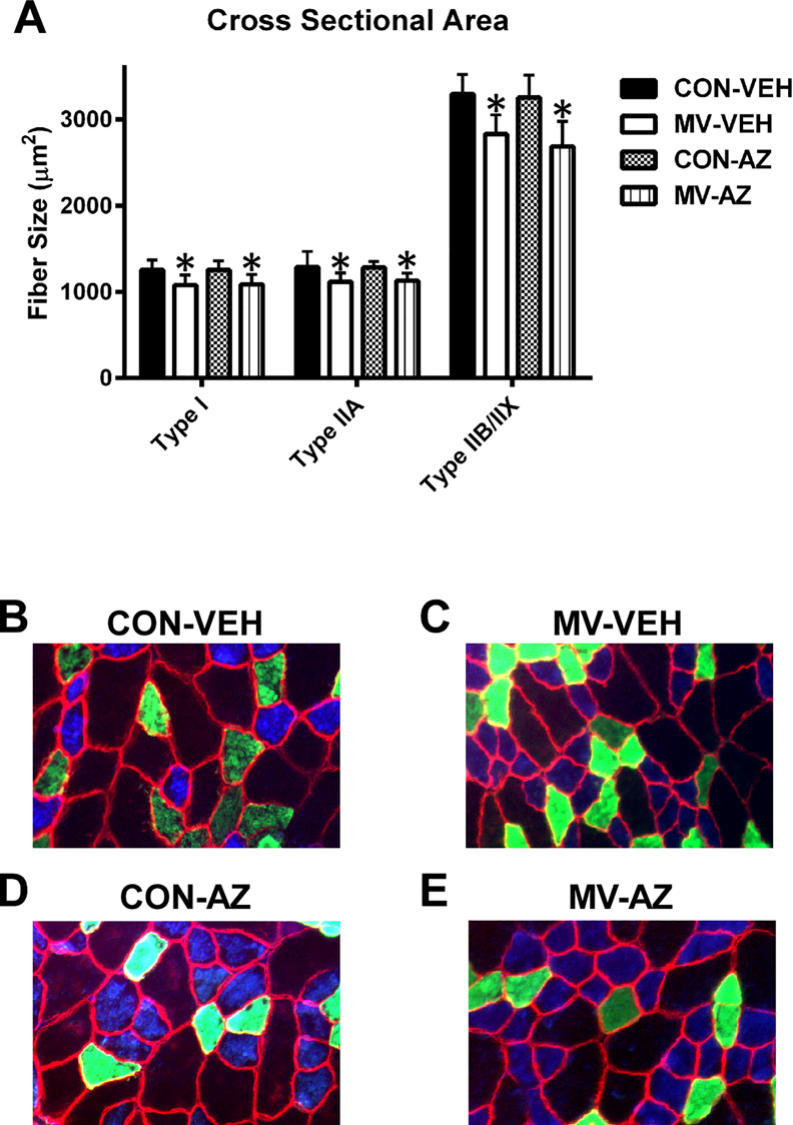
Cross-sectional area and representative images. A) Quantification of diaphragm muscle fiber cross-sectional area (CSA) by fiber type. Values are mean ± SEM. * = main effect for MV (*p* < .05). B-E) Representative staining of MHC I (blue), MHC IIa (green), and dystrophin (red) proteins B) CON-VEH C) MV-VEH D) CON-AZ E) MV-AZ, n = 6-9/group.

Similar to previous reports, 12 hours of MV led to significant decreases in the CSA of Type I, Type IIA, and Type IIB/IIX diaphragm muscle fibers (Fig 4). AZ treatment did not attenuate these MV-induced decreases in diaphragm fiber size. Therefore, blockage of the RyR1 during MV is not sufficient to prevent MV-induced diaphragm atrophy and suggests that leakage of calcium through the RyR1 is not responsible for MV-induced diaphragmatic atrophy.

## Discussion

### Overview of major findings

These experiments tested the hypothesis that pharmacological blockade of the RyR1 in diaphragm fibers with AZ would prevent MV-induced increases in mitochondrial ROS production, protease activation, and diaphragmatic atrophy. Nonetheless, our results do not support this proposition because treatment with AZ did not protect against the MV-induced increase in mitochondrial ROS emission or protease activation in the diaphragm. Moreover, AZ treatment (i.e., pharmacological inhibition of the RyR1) does not protect against MV-induced diaphragm fiber atrophy. A discussion of these and other important findings follows.

### Azumolene is sufficient to block RyR calcium release but does not prevent MV-induced diaphragmatic atrophy

Increased mitochondrial ROS production has been established as a cause of VIDD [10]. Although controversy exists, it is widely reported that mitochondrial uptake of calcium results an increase in mitochondrial ROS production [27]. It appears likely that prolonged MV results in increased cytosolic levels of free calcium because MV results in activation of the calcium-activated protease calpain in the diaphragm [40]. We reasoned that leakage of calcium from the SR via the RyR1 is a potential mechanism to explain the MV-induced increase in cytosolic calcium levels in diaphragm fibers and the current experiments were designed to test this prediction.

It is well established that AZ is a successful antagonist of RyR1 opening, and our contractile force measurements on both the soleus and diaphragm muscles confirm these findings [28–30]. Importantly, the observed decrease in muscle force production confirms that AZ was taken up by the diaphragm and that blockade of the RyR1 occurred [41]. However, AZ treatment failed to prevent MV-induced increases in mitochondrial ROS emission and activation of the proteases calpain and caspase-3, which are known requirements for MV-induced muscle wasting [10,12]. Thus, our findings that RyR1 blockade did not protect against diaphragmatic atrophy during prolonged MV are not surprising, as AZ failed to prevent several known upstream mediators of VIDD.

In an interesting coincidental finding, we report for the first time that 12 hours of MV induces a significant decrease in soleus muscle specific force production. Our group has previously reported that the plantaris muscle does not atrophy following 12 hours of mechanical ventilation, but the observation that prolonged MV results in a rapid decline in limb muscle force production is a new finding [34]. Our current data are in contrast with a previous study demonstrating that maximal tetanic force production in the soleus muscle remains unchanged following 48 hours of MV [42]. A potential explanation for this variance between studies is that 12 hours of MV did not result in a decrease in soleus muscle mass whereas 48 hours of MV decreased soleus mass by 20%. Thus, following 48 hours of MV, the soleus muscle may have reached a new steady state of specific force production.

### Experimental Limitations

Similar to all investigations, the current experiments have experimental limitations. For example, in our control animals, both the AZ treated (CON-AZ) and VEH-treated (CON-VEH) animals were sacrificed immediately following infusion of AZ or VEH, instead of remaining anesthetized for 12 hours. The reason for this experimental approach is that our preliminary experiments revealed animals treated with AZ stopped breathing and therefore, these animals were unable to maintain adequate alveolar ventilation on their own to survive for 12 hours without ventilator assistance. As such, our AZ animals received only an acute dose of AZ before sacrifice, while the MV-AZ animals received a constant infusion of AZ.

It is also possible that AZ treatment will not prevent all forms of calcium release from the RyR1. Specifically, while it is well-documented that AZ blocks electrical stimulation-induced calcium release from the SR, it is unclear if AZ protects the RyR1 against oxidation-induced calcium release. In this regard, it is established that oxidized RyR1 in skeletal muscle are depleted of calstabin1, a RyR1 binding protein required for normal RyR1 function [17]. Indeed, oxidation of RyR1 in skeletal muscles results in leaky channels and muscle weakness [17,18]. Data presented in abstract form by Matecki et al. reveal that prolonged MV leads to oxidation of the RyR1 and that maintaining the interaction between the RyR and calstabin1 is sufficient to prevent VIDD (Am J Respir Crit Care Med 187;2013:A3019; Am J Respir Crit Care Med 189;2014:A3885). Additional experiments will be required to determine if the ability of maintaining the interaction between the RyR1 and calstabin1 occurs by preventing calcium leak or through another mechanism.

### Conclusions

These experiments demonstrate that treatment of animals with AZ is not sufficient to prevent the MV-induced activation of calpain or caspase-3 or prevent MV-induced atrophy of diaphragm muscle fibers. Therefore, these findings do not support the hypothesis that the release of calcium through the RyR1 is responsible for increased mitochondrial ROS production, protease activation, and atrophy in the diaphragm following 12 hours of MV.

## Supporting Information

S1 File Western Blots. All lanes of Western blots in Fig 3 are presented in Supporting Information.
(PDF)
