# Supplementary material for: Blockage of the Ryanodine Receptor via Azumolene Does Not Prevent Mechanical Ventilation-Induced Diaphragm Atrophy
Source: PLoS One. 2016 Feb 5;11(2):e0148161. doi: 10.1371/journal.pone.0148161 (PMC4744044; doi:10.1371/journal.pone.0148161)

Western Blot for Calpain-1
Samples Alternate CON-VEH MV-VEH CON-AZ MV-AZ

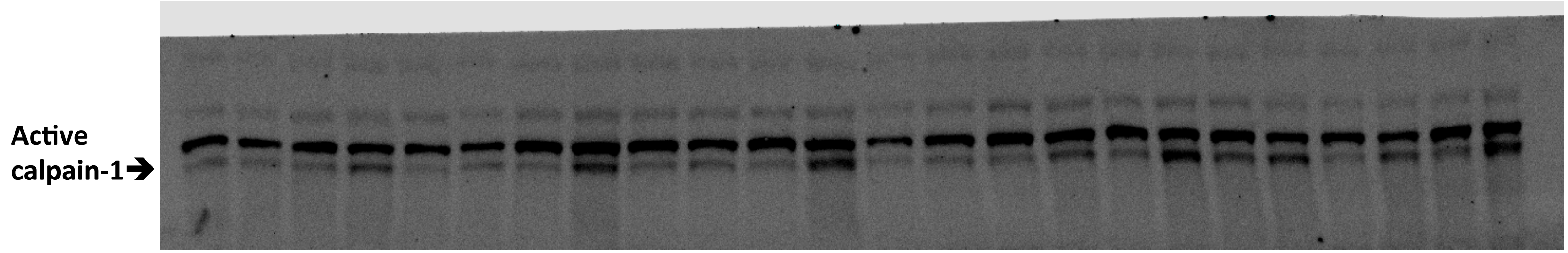

α-tubulin normalization for Calpain-1
Same membrane as above

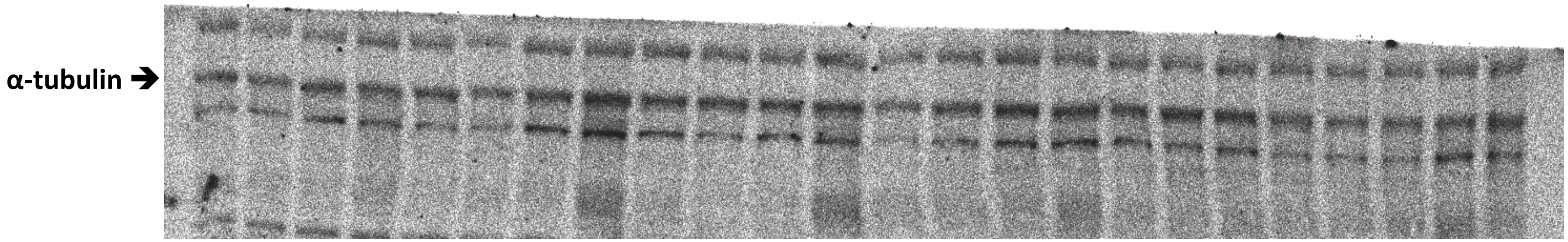

Western Blot for α-II-Spectrin
Samples Alternate CON-VEH MV-VEH CON-AZ MV-AZ

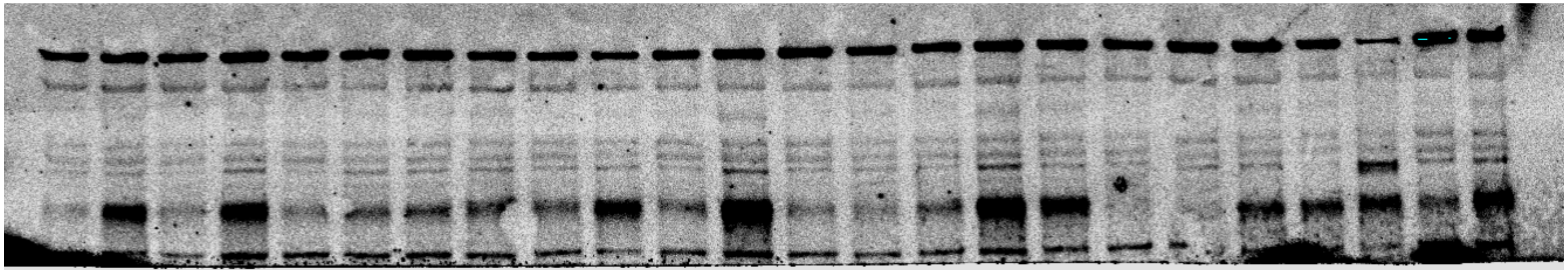

α-tubulin normalization for α-II-Spectrin
Same membrane as above

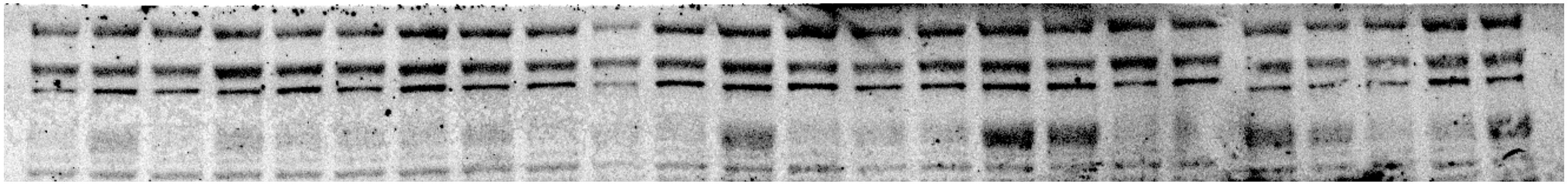

Supplement: S1 File — All lanes of Western blots in Fig 3 are presented in Supporting Information. (PDF) [file pone.0148161.s001.pdf]
